# Subjective and Quantitative Measurement of Wavefront Aberrations in Nuclear Cataracts – A Retrospective Case Controlled Study

**DOI:** 10.4103/0974-9233.48858

**Published:** 2009

**Authors:** Upender K. Wali, Alexander A. Bialasiewicz, Nadia Al-Kharousi, Syed G. Rizvi, Habiba Baloushi

**Affiliations:** 1 From the Department of Ophthalmology, College of Medicine and Health Sciences, Sultan Qaboos University, Muscat, Oman; 2 From the Dept. of Family Medicine and Public Health; College of Medicine and Health Sciences, Sultan Qaboos University, Muscat, Oman

**Keywords:** Aberrometry, Nuclear Cataract, Zernike Polynomials

## Abstract

**Purpose:**

To measure, quantify and compare Ocular Aberrations due to nuclear cataracts.

**Setting:**

Department of ophthalmology and school for ophthalmic technicians, college of medicine and health sciences, Sultan Qaboos University, Muscat, Oman.

**Design:**

Retrospective case controlled study.

**Methods:**

113 eyes of 77 patients with nuclear cataract (NC) were recruited from outpatient clinic of a major tertiary referral center for Ophthalmology. Patients having NC with no co-existing ocular pathologies were selected. All patients were subjected to wavefront aberrometry (make) using Hartmann-Shack (HS) aberrometer. Consents were taken from all patients. Higher order Aberrations (HOA) were calculated with Zernike polynomials up to the fourth order. For comparison 28 eyes of 15 subjects with no lenticular opacities (control group) were recruited and evaluated in an identical manner. No pupillary mydriasis was done in both groups.

**Results:**

Total aberrations were almost six times higher in NC group compared to control (normal) subjects. The HOA were 21 times higher in NC group, and coma was significantly higher in NC eyes compared to normal (control) group. The pupillary diameter was significantly larger in control group (5.48mm ± 1.0024, p<.001) compared to NC (3.05mm ± 1.9145) subjects (probably due to younger control age group). Amongst Zernike coefficients up to fourth order, two polynomials, defocus (Z_2_^0^) and spherical aberration (Z_4_^2^) were found to be significantly greater amongst NC group, compared to normal control group.

**Conclusion:**

Nuclear cataracts predominantly produce increased defocus and spherical aberrations. This could explain visual symptoms like image deterioration in spite of normal Visual acuity.

Cataract is the leading cause of preventable blindness worldwide.^1^ While assessing VA we measure the limit of resolution with high contrast figures like Snellen's optotypes in a glare free room which does not represent normal everyday situation in the life of an elderly. There may be high disparity between subjective visual disturbance and objective VA measurements. Little has been documented about the correlation between objective measurement of VA and subjective visual disturbance. Elliot et al have compared clinical tests of visual function in cataract with patients' perceived visual disability.^2^ In early nineties, cataract symptom scale and catquest questionnaires were implemented to assess functional limitations of patients with cataract,^3^–^4^ however, neither were of use in clinical practice due to time factor. Wavefront aberrometry was introduced in ophthalmology practice to objectively evaluate visual performance. Wavefront analyzer is used to evaluate the quality of an optical system in today's high-tech world. Wavefront is the pack of light beam or bundle of rays reflected from the retina and coming out of the eye which is then assayed by the sensor called Shack-Hartmann wavefront sensor (originally developed for high-energy laser and astronomy applications). The light used in the measurement is in the near infrared region of the spectrum where the eye is nearly insensitive. The sensor has lenslets which divide the wavefront into pieces. More the lenslets more is the resolution, and the quality of detecting HOA. The Hartmann-Shack aberrometer has 1452 sensors (lenslets). This array of lenslets is connected to a CCD camera.

Aberrometry does not provide direct imaging of the eye; it provides a picture of the eye's optical quality. Corneal refractive surgery is one of the fields where this technology has contributed a lot.^5^

This study was undertaken to determine whether wavefront aberrometry can be used as a tool in eyes with nuclear cataracts to assess their visual function, and thereby, help cataract surgeons as well as patients in predicting quality of visual outcome.

Aberration means a deviation from the proper or expected course, a defect of focus such as blurring or distortion in an image. It may be a physical defect in the optical element, as in a lens, that causes such imperfection. Scattering of light,^6^ optical aberrations (measured in micrometers) and loss of contrast sensitivity are main causes of image degradation in nuclear cataract patients. Hartmann-Shack (HS) aberrometer was developed to measure the optical aberrations in the whole eye.^7^ The Hartmann-Shack aberrometer (which is programmed) reconstructs the wavefront. This wavefront has a shape and amplitude. Zernike (Fritz Zernike from Amsterdam, won Nobel Prize in Physics in 1953) polynomials help converting this complex shape and amplitude of the wavefront into a series of simple mathematical figures. Aberration table is the most important data display in the program.

Spheres and cylinders are not the only refractive errors of the eye. Additional refractive errors known as higher order aberrations (HOA) also exist. These additional refractive errors are normally so small that they have little effect on routine vision. In summary HOA are the refractive errors beyond spheres and cylinders. They are important because patients with such aberrations may experience uncomfortable visual symptoms even when they have six-by-six vision.

While cortical opacification is measured objectively by determining the percentage of lens that is opaque, nuclear opacification is determined by measuring the optical density (Lens opacities classification III [LOCS III].^8^

## MATERIAL AND METHODS

Wavefront Aberration Supported Corneal Ablation (WASCA) system analysis is a class 1 laser device. Written consent was obtained from all patients. Being a non-interventional study, the approval from Medical Research Ethics Committee was not required. Patients were selected on the basis of clinically observable nuclear cataract with no co-existing ocular pathology. A standard ophthalmic assessment was performed in all patients. This included best corrected visual acuity (BCVA) using Snellen chart at six meters, Slit-lamp biomicroscopy (Haag-Streit, Switzerland), and intraocular pressure measurement (Goldmann). Aberrometry was performed by a single trained senior technician (HB) using HS Aberrometer (Zeiss Asclepion Germany). Picture acquisition was done by dimming the room light and target light so that maximum diameter of the patient pupil could be achieved without using a mydriatic. The fellow eye was provided with a target at a distance of 4 meters to avoid accommodation. Where auto-refraction failed to converge (as happened in few young patients with good accommodation), a manual refraction in the aberrometer was performed. The measurements were repeated at least three times or till a well focused image of the eye was obtained. All corneal contact procedures that might have affected the corneal epithelium or tear film (like applanation tonometry) were done after the aberrometry. The root mean square (RMS) used for analysis was computed by Zywave.

For comparison 28 eyes of 15 subjects with no clinical detectable cataract or any other ocular pathology, except refractive errors, were recruited and served as a control group. These subjects were mainly selected from the staff members of the hospital, or relatively young relatives of the patients. All control eyes were subjected to similar psycho-optical tests and procedures as eyes with NC, except A-scan and applanation tonometry which were not done in control eyes.

The exclusion criteria were: (1). A Hartmann image that could not be analysed automatically. (2). Eyes with associated corneal, vitreous and retinal pathologies. Normal eyes in control group did not have any ocular disorder.

Zernike modes or aberrations are labeled as double index scheme (Z_2_≤). The subscript 2 denotes the Zernike order (Z_2_ means second order, Z_3_ means third order) while the superscript (≤) means the mode or the aberration within that order.

The HOA were measured up to fourth order Zernike polynomials without dilating the pupil. From the Zernike coefficients, we calculated defocus (Z_2_), cartesian astigmatism (Z_2_^2^), trefoil of cosine phase (Z_3_^3^), Coma like aberrations (third order component (Z_3_), tetrafoil of cosine (Z_4_^4^), tetrafoil of sine phase (Z_4_^−4^), and spherical aberrations (Z_4_). Corneal topography was measured simultaneously using placido disk attached to the HS wavefront sensor.

## RESULTS

**Figure 1:**
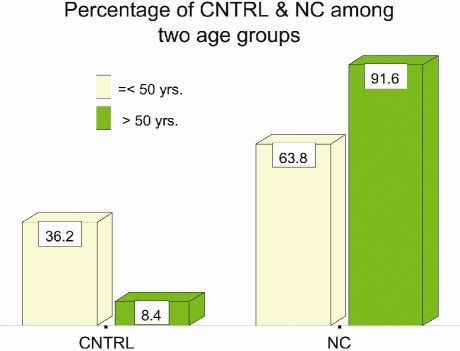
Percentage of two age groups amongst nuclear cataract (NC) and control group

Table 1: Mean age of the patients with NC (n=113) was 58 years; range 22 years to 81 year. The control group had a younger mean age of 44.5 years; range 18 years to 66 years. Amongst NC group 58.4% were males, while in control group females dominated with 67.86%. Mean age for males in NC group (n=66) was 60.7 years, and for females (n=47) 54.3 years. In the control group mean age for males (n=9) was 40.2 years, and for females (n=19) 46.5 years. The percentage of age groups between NC and control group was significant (p<.001) (Fig. 1).

**Table 1:** Average Age of the Individuals among Control and Nuclear Cataract Groups and Gender Difference

	Total	Males	Females	Significance (Male vs. Female)
CNTRL*	44.46 ± 13.86	40.22 ± 16.82	46.47 ± 12.21	P = .273

NC§	58.05 ± 10.71	60.73 ± 9.49	54.30 ± 11.28	P = .001

Significance (CNTRL vs. NC)	P < .001	P < .001	P = .015	

* control

§ nuclear catarct

**Figure 2:**
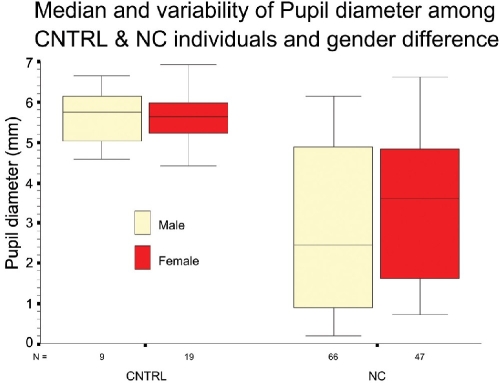
Mean pupil diameter in control versus nuclear cataract group, and in two genders

The mean pupil diameter (Fig 2; Table 2) for control group was 5.48mm (SD ±1.0024) versus 3.05mm (SD ±1.9145) for NC group. This difference was found to be significant (p<.001). All Zernike prescriptions must include pupil size. Same eye may have different set of coefficients for different pupil sizes because aberrations become more pronounced as the peripheral aspects of the eye's optical system are uncovered (dilated pupil).

**Table 2:** Comparison of Pupil Diameter, Total Aberrations, Higher Order Aberrations, and Coma between Nuclear Cataract and Control Groups

Diagnosis	Median	Mean	Std. Deviation	Significance
Pupil diameter (mm)				
CNTRL (28)	5.695	5.850	1.0024	P <.001
NC (113)	3.120	3.0510	1.9145	

Total aberr				
CNTRL (28)	1.530	1.7364	1.0752	P = .262
NC (113)	1.680	11.6011	24.2214	

HOAs				
CNTRL (28)	.260	.3150	.1451	P = .003
NC (113)	.440	6.7900	15.7432	

Coma				
CNTRL (28)	1.170	1.5018	1.1315	P <.001
NC (113)	2.590	20.2052	54.3304	

The mean values for ocular and corneal wavefront aberrations were determined in both NC and control groups. Mann-Whitney U test (non-parametric) was applied as the distribution did not follow a normal pattern.

Mean Total aberrations (HOA plus Lower order aberrations) were six times larger (11.6011 SD ±24.2214) in NC eyes than in control eyes (1.7364 SD ±1.0752), however, this difference was not found to be significant, probably due to younger age group of the control eyes. Mean HOA for NC group (n=113) was 6.7900 SD ±15.7432) compared to.3150 (SD ±.1451) for control group (n=28). This difference was significant (p=.003). Coma in NC group was almost 13 times higher (20.2052 SD ±54.3304) than in control group (1.5018 SD ±1.1315). This too was statistically significant (p<.001) (Table 2).

Table 3. The difference between the NC and the control groups was found to be significant in two aberrations, namely defocus (Z_2_^0^ p<.001, mean for NC group being - 28.4146 SD ± 54.9631) and spherical aberration (Z_4_^2^ p<.045, mean for NC group being.45652 SD ± 18.16863). Aberrations like Cartesian astigmatism (Z_2_^2^), triangular astigmatism (Z_3_^−3^), coma of sine phase (Z_3_^−1^), spherical aberration (Z_4_^−2^) were found to be higher in NC group but not significantly.

**Table 3:** Comparison of Zernike Coefficients up to Fourth Order between Nuclear Cataract and Control Group

Diagnosis	Median	Mean	SD	Significance
Z (2, −2) CNTRL (28)	.2050	.4732	1.3215	P = .547
NC (113)	9.000E-02	.4715	2.7603	

Z (2, 0) CNTRL (28)	.2450	−.2332	3.2588	P = .001
NC (113)	−1.9700	−28.4146	54.9631	

Z (2, 2) CNTRL (28)	.2050	−.1368	2.4110	P = .06
NC (113)	.8400	.6885	4.9149	

Z (3, −3) CNTRL (28)	1.50E−03	6.61E−02	.33687	P = .871
NC (113)	2.20E−02	084883	7.44165	

Z (3, −1) CNTRL (28)	−4.8E−02	−1.7E−02	.26977	P = .320
NC (113)	9.90E−02	1.86118	12.64040	

Z (3, 1) CNTRL (28)	−1.4E−02	7.95E−02	.56771	P = .554
NC (113)	−2.8E−02	−2.28796	14.58168	

Z (3, 3) CNTRL (28)	−.21550	−.13079	.62806	P = .535
NC (113)	−7.6E−02	−.17025	3.74995	

Z (4, −4) CNTRL (28)	1.15E−02	4.65E−02	.18904	P = .561
NC (113)	1.20−02	−.12461	3.13413	

Z (4, −2) CNTRL (28)	5.10E−02	4.91E−02	.12220	P = .979
NC (113)	1.90E−02	2.91361	13.88319	

Z (4, 0) CNTRL (28)	−2.3E−02	−2.5E−02	.20396	P = .105
NC (113)	−.12600	−11.98452	34.11593	

Z (4, 2) CNTRL (28)	−.12950	−.12218	.15934	P = .045
NC (113)	1.70E−02	.45652	18.16863	

Z (4, 4) CNTRL (28)	−3.0E−02	−8.4E−02	.21417	P = .800
NC (113)	−5.6E−02	−1.53828	9.17589	

CNTRL = control; NC = nuclear cataract; HOA = higher order aberration; N = number; Z = Zernike coefficient; NS = not significant; P = p value

Zernike coefficients like astigmatism with axis (Z_2_^−2^) coma of cosine phase (Z_3_^1^), trefoil of cosine phase (Z_3_^3^), spherical aberration (Z_4_^0^), tetrafoil of cosine phase (Z_4_^4^) and tetrafoil of sine phase (Z_4_^−4^) were found to be higher in control group compared to NC group; however, the measurements were not statistically significant (Table 3).

In the NC group, the mean ocular spherical aberration showed much higher negative polarity, yielding sufficient reproducibility (−11.984 SD ± 34.115) versus only −.025 SD ±.203 for control group. Negative polarity in spherical aberration in NC has also been reported by Kuroda et al.^9^ The spherical like aberration was greater than the coma-like aberration, which was consistent with the symmetrical nature of the NC (Table 3).

### POLARITY:

**NC Group:** Amongst 12 Zernike coefficients, six aberrations (Z_2_^−2^, Z_2_^2^, Z_3_^−3^, Z_3_^−1^, Z_4_^−2^, Z_4_^2^) showed positive polarity while other six (Z_2_^0^, Z_3_^1^, Z_3_^3^, Z_4_^−4^, Z_4_^0^, Z_4_^4^) showed negative polarity. The negative polarity means the wavefront in the papillary area is relatively delayed, indicating a myopic shift (Table 3).

**Control Group:** Five aberrations (Z_2_^−2^, Z_3_^−3^, Z_3_^1^, Z_4_^−4^, Z_4_^−2^) out of 12 Zernike coefficients showed positive polarity, while other seven aberrations (Z_2_^0^, Z_2_^2^, Z_3_^−1^, Z_3_^3^, Z_4_^0^, Z_4_^2^, Z_4_^4^) showed negative polarity (Table 3).

## DISCUSSION

Wavefront technology has immensely contributed to the measurement and quantification of HOA in human eyes. Aberrometers use a new measuring principle for the objective determination of the visual performance of the eye. It provides a complete analysis of the refractive path of the eye. Clinically this technology has been used to measure and quantify aberrations induced by keratoconus,^10^ and currently is being used widely in customizing laser refractive surgery.^11^ Posterior corneal surface contributes approximately 13% to the aberration status of the whole eye. The use of wavefront technology to measure and quantify cataract induced aberrations has been limited.

Kuroda et al reported negative spherical like aberration, defined as 4^th^-order component (S_4_) and 6^th^ order component (S_6_) in a single case of nuclear cataract.^12^ This is compatible with the negative fourth order (Z_4_^0^) component in our patients with NC, though our series had larger number of cases. In a latter study by the same authors, they found that nuclear opacities always induced negative spherical aberration which is confirmed by our study as well,^9^ (Table 3). Though Kuroda et al used polynomials for 7.0mm pupil against 3.05mm in our cases; the aberrometers used follow the same optical principle. Patients in our study revealed that eyes with primarily NC exhibited significantly higher spherical aberration for Z_4_^2^ polynomial than eyes in control group (p<.045). Although increase in other spherical aberration components (Z_4_^−4^, Z_4_^0^, Z_4_^4^, Z_4_^−2^) was not statistically significant, we believe the trend followed due to pupil not being dilated in our cases.

Study by Nisha Sachdev et al has also reported statistically significant spherical aberration in nuclear opacification compared to normal human eyes.^13^ Same authors have reported three times greater HOA in nuclear opacification compared to control eyes. In comparison, our study exhibited 21 times more HOA in nuclear cataracts compared to control eyes (p<.003). This large difference could be due to larger pupil (6mm) in their study compared to 3.05mm in our patients. For WASCA there is no need to dilate the pupil, patient should be sitting up in a dark room and a distant target should be shown to relax the accommodation.

Also since our cataract grading was not based on pentacam or LOCS scale, the difference in the density of NC between two groups could be the reason for such a large difference. The increase in total aberrations (TA) and coma in our series is well documented by Nisha Sachdev et al.^13^ some of the variations in HOA could have been due to other variations like Lower Order Aberrations (spherical and cylindrical refractive errors and age differences.^14^ Second-order aberrations or lower-order aberrations are spherical defocus (myopia or hypermetropia) and astigmatism. The second-order modes or aberrations are the ones which we routinely correct with spectacles or contact lenses and non-wavefront LASIK.

Total aberrations (11.601 in NC group versus 1.736 in control group in our study) increase in eyes with cataract due to local refractive changes in the lens. This has been observed by Kuroda et al^9^ as well, although the authors in their study mainly focused on difference between corneal total HOA and ocular total HOA. The negative polarity of spherical aberration (Z_4_^0^) in nuclear cataracts in our patients is strongly corroborated by similar observation by the same authors (Kuroda et al). This suggests delay of wavefront when the ray travels inside a hard nucleus with increased refractive index. Such negative polarity of spherical aberrations (wavefront delay) is well consistent with the slower velocity measurements of extracted senile nuclear cataractous lenses using ultrasound, than in normal lenses.^15^ However, few previous reports have shown positive polarity in ocular spherical aberration in elderly patients.^14^ The weak negative polarity of spherical aberration (Z_4_^0^) in our control group could be due to relatively younger age group (mean 44.46 years). For the sake of interest, polarity is usually positive in cortical cataracts and negative in NC.

The limitations of our study were that LOCS III system for cataract grading was not possible, and the use of Pentacam Scheimpflug photography for in-vivo measurements and quantification of lens density would have been more scientific; however, a recent study has shown that LOCS III criterion as an economic cataract grading system provides data that are in satisfactory concordance with the results obtained by Pentacam Scheimpflug system.^16^

Until early nineties there was no way to clinically measure such aberrations, or even if HOA were significant and measurable, there was no practical way to correct them. HS aberrometer with Scheimpflug camera^16^ revolutionalized the concept of such aberrations.

In conclusion, wavefront technology is a unique tool for measuring and quantifying aberrations produced by the eye as a whole optical system. HS aberrometer measures the deterioration of image quality objectively and quantitatively in eyes with cataract. The results suggest that nuclear cataracts induce high spherical aberrations and varying amounts of tetrafoil.
